# Intra-retinal Arterial Cannulation using a Microneedle for Central Retinal Artery Occlusion

**DOI:** 10.1038/s41598-018-19747-7

**Published:** 2018-01-22

**Authors:** Kazuaki Kadonosono, Shin Yamane, Maiko Inoue, Tadashi Yamakawa, Eiichi Uchio

**Affiliations:** 1 Department of Ophthalmology and Micro-technology, Yokohama City University, Yokohama, 232-0024 Japan; 2 Department of Internal Medicine, University of Yokohama City University, Yokohama, 232-0024 Japan; 3 Department of Ophthalmology, Fukuoka University, Fukuoka, 814-0180 Japan

## Abstract

Central retinal artery occlusion (CRAO) is a severe disease, often causing blindness. We evaluated the efficacy and safety of a surgical procedure for the treatment of acute CRAO in which retinal arterial cannulation with tissue plasminogen activator (tPA) is performed. The surgical procedure consisted of vitrectomy followed by cannulation of the central retinal artery and injection of tPA (200 μg) using a 47-gauge microneedle. Thirteen CRAO patients were treated within 48 hours of the onset of symptoms. The central retinal artery of all 13 eyes was successfully cannulated. The mean interval between the onset of symptoms and surgery was 38.7 hours. The results for all 13 eyes treated showed a statistically significant improvement in mean visual acuity between before and one month after treatment (−1.60 vs. −0.82 logarithmic values for minimum angle resolution (LogMAR), p = 0.0021). Fluorescein angiography showed complete reperfusion and incomplete reperfusion in 10 eyes and 3 eyes, respectively. Recently developed surgical instruments have made retinal-arterial cannulation feasible. Intra-retinal-arterial cannulation has potential as a method of improving visual function and microcirculation in eyes affected by CRAO.

## Introduction

Central retinal artery occlusion (CRAO) is caused by a thrombus or embolism in the central retinal artery, usually in the vicinity of the optic nerve head. It is considered an ophthalmological emergency, often causing blindness due to the resulting inner retinal ischemia^1,2^. The incidence of acute CRAO is approximately 1/10,000 population, and 92% of patients are left with a counting finger visual acuity or less^3,4^. Since significant associations have been found between CRAO and both atherosclerotic pathology and cardiac abnormalities, the number of patients with the disease is expected to increase, particularly in aging societies^5^.

A recent study showed that the most common cause of CRAO is embolism. In 71% of CRAO patients the ipsilateral internal carotid artery contains plaque, made up of cholesterol or platelet-fibrin. Pieces of this plaque can cause blockages in the central retinal artery leading to development of CRAO^6^. During the past decade both intravenous thrombolysis and intra-arterial thrombolysis with tissue plasminogen activator (tPA) have been used to treat CRAO^7–15^. It seems that the efficacy of intra-vessel fibrinolysis as a treatment for CRAO remains controversial. Cannulation of the central retinal artery was reported in one case to be an effective surgical treatment, allowing direct mechanical access to the site of the obstruction^7^. However, there have not been any reports on retinal arterial cannulation since then because this surgical procedure has been thought to be challenging.

We have developed an endovascular cannulation procedure that enables treatment of CRAO by injecting a liquid to dislodge the embolus in the central retinal artery using a recently developed 47-gauge microneedle^16,17^. Here we report a prospective study on endovascular cannulation of the central retinal artery with injection of tPA.

## Methods

### Patients

The Institutional Review Board of Yokohama City University Medical Center approved this study, and it was conducted in accordance with the tenets of the Declaration of Helsinki (UMIN000022972, registered at July 1^st^, 2016). After all of the patients had provided written informed consent, they were scheduled for cannulation using tPA injection. The study was conducted between July 2016 and May 2017 in the Department of Ophthalmology and Micro-technology at Yokohama City University.

### Eligibility

CRAO was diagnosed by performing fluorescein angiography and a funduscopic examination to classify. CRAO is classified into three stages: incomplete, subtotal, and total. Incomplete CRAO is characterized by mild retinal edema, slightly decreased visual acuity, and unclear cherry red spot. Subtotal CRAO is a typical CRAO disease and is characterized by narrowed retinal arteries in all four quadrants and ischemic retinal edema associated with a cherry-red spot at the center of the macula. Total CRAO is so severe as to present almost no light perception. The inclusion criteria were CRAO with initial best-corrected visual acuity better than hand motion, but not higher than 20/40 and onset of symptoms less than 48 hours prior to the initial consultation. The exclusion criteria were a coagulation disorder, recent stroke, severe arterial hypertension, a bleeding disorder, head trauma, recent gastrointestinal bleeding, proliferative diabetic retinopathy, hypertensive retinopathy and eyes with arteritis.

### Examinations

The preoperative evaluation included a standard ophthalmic examination, determination of best-corrected Snellen visual acuity (BCVA), and optical coherent tomography (OCT) examination before surgery. Wide-field fluorescein angiography was performed preoperatively, and again at 3 days and one-week post-surgery to determine the arm-retina time and whether retinal vessel reperfusion was complete. Each patient’s chart contained the following data: age; gender; baseline best-corrected Snellen visual acuity and logarithmic minimum angle resolution values (logMAR); best-corrected Snellen visual acuity and logMAR values at one week and one month postoperatively; and interval between the onset of symptoms and surgery. Every patient underwent general examinations that included ipsilateral carotid color Doppler ultrasound examination and an echocardiogram to identify internal carotid artery plaques and cardiac valve diseases (Tables 1 and 2).

**Table 1:** Demographic and Clinical Characteristics in CRAO.

No. of patients	Mean age,(range)	Male/female	Time from symptom onset	CRAO Stages		
13	69 ± 11.2 (58–83)	10/3	37.8 (26–48 hrs.)	Incomplete (%)	Subtotal (%)	Total (%)
				1 (8%)	11 (77%)	1 (15%)

CRAO = central retinal artery occulusion.

**Table 2:** Fluorescein angiography, Carotid Doppler and Echocardiogram Findings in CRAO.

Perfusion 3days after cannulation		Carotid Doppler				Echocardiogram		
Incomplete (%)	Complete (%)	Occlusion (+) (%)	Occlusion (−) (%)	Plaque (+)(%)	Plaque (−) (%)	Normal (%)	Abnormal, no embolic source (%)	Abnormal, with embolic source (%)
3 (21%)	10 (79%)	10 (77%)	3 (13%)	12 (92%)	1 (8%)	13 (100%)	0	0

CRAO = central retinal artery occulusion.

### Surgical technique

Surgery was performed under local anesthesia with systolic blood pressure maintained at less than 120 mmHg by administering antihypertensive drugs to minimize intraoperative bleeding.

**Figure 1:**
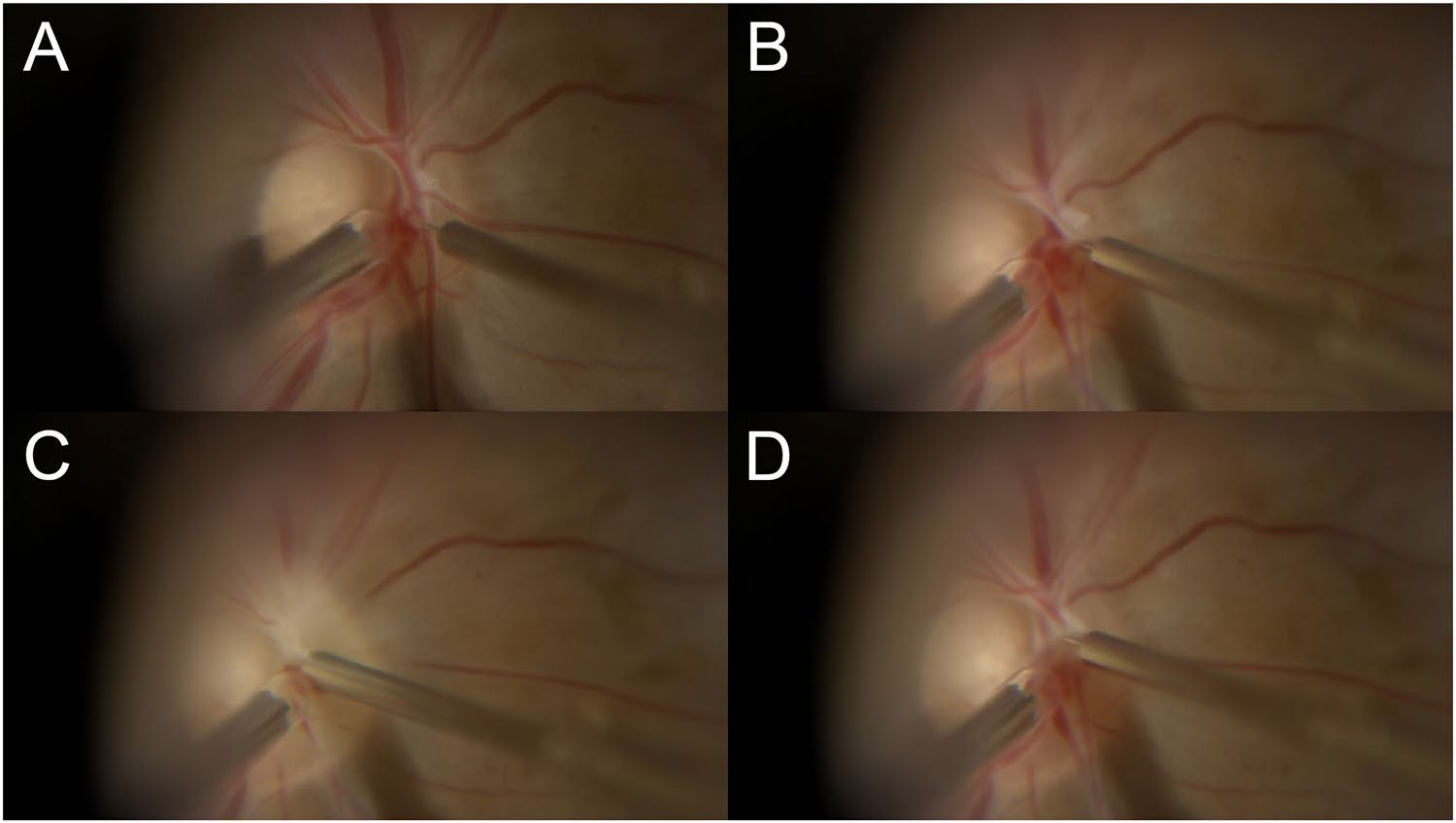
Surgical procedure for retinal-arterial fibrinolysis to treat eyes with central retinal arterial occlusion with a microneedle. The outer diameter of the microneedle is 50 micrometers although it appears larger in the intraoperative photograph due to perspective. It is held in the right hand and inserted into the central retinal artery while holding a soft-tip needle in the left hand to stop any bleeding (**A**). Tissue plasminogen activator solution is injected into the vessel through the microneedle (**B**). The arterial vessel turns white in response to the pressure of injected solution (**C**). The solution is injected over a 3-minute period, and the needle is removed gently (**D**).

The microneedle used has a 50 μm outer diameter and 20 μm inner diameter, and is made of stainless steel^16,17^ (Fig. 1). This needle is estimated at 47-gauge. After performing the core vitrectomy, the needle was connected to the 10-cc syringe of the viscous fluid control unit of the vitrectomy machine (Constellation^®^, Alcon Lab Inc., Dallas-Fort Worth, TX, USA) and used to slowly inject a 0.4 mL volume of tPA (Monteplase, Eisai Co., Tokyo, Japan) solution containing 50.0 μg/0.1 mL (total dose: 200 μg). A soft tip cannula enables aspiration of any blood lost at the puncture site and allows improved visualization. The total volume of the tPA solution was accurately injected into the central retinal arterial vessel at a pressure of 30 psi over the course of approximately 3 minutes. The procedure is shown in Fig. 1 (Video).

The microneedle was then removed and followed by a fluid-air exchange, and the patient was instructed to remain prone overnight to prevent postoperative bleeding from the damaged central artery of the retina. All patients took aspirin (50 mg) daily as an anti-platelet drug after surgery.

### Statistical Analysis

Preoperative and 1-week and 1-month postoperative visual acuity values were statistically analyzed for significant differences by performing the Wilcoxon signed-rank test. Statistical significance was assigned at a *P* value of <0.05.

## Results

### Demographic and Clinical Characteristics

There were 13 subjects (3 female and 10 male), and their mean age was 71 years (range, 57–83 years). After consultation all patients agreed to undergo the proposed surgical treatment. The diagnoses were incomplete CRAO in one eye, subtotal CRAO in 11 eyes, and total nonarteritic CRAO in one eye. Cilioretinal arteries were absent in all patients treated in the study.

The average interval between the onset of symptoms and surgery was 37.8 hours (range, 26–47 hours). Carotid Doppler ultrasound showed plaques in 10 (77%) of the 13 eyes. Echocardiography showed no evidence of any heart valve disease (Table 1).

### Visual Outcome

**Figure 2:**
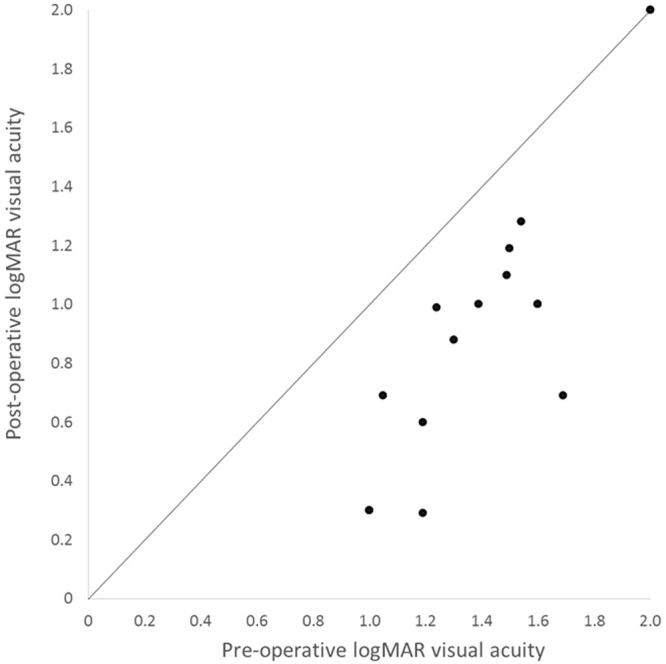
Change in visual acuity before and one month after surgery. Visual acuity improved in 12 of 13 eyes (91%) after surgery more than 0.3 LogMAR. LogMAR = logarithm of the minimum angle of resolution.

Preoperative visual acuity in the affected eyes ranged from 20/200 to counting fingers. One week after cannulation, visual acuity in 11 (85%) of the 13 eyes had improved by more than 0.3 LogMAR, but there was no change in visual acuity in the other 2 eyes (15%). One month after cannulation, visual acuity in 12 (92%) of the 13 eyes had improved by more than 0.3 LogMAR, but it remained unchanged in the remaining eye (8%, Fig. 2).

The results showed that mean Snellen visual acuity in the 13 eyes had improved from 20/800 at baseline to 20/200 at one week and to 20/130 at one month, and the difference between LogMAR visual acuity preoperatively and at one month postoperatively (−1.6 vs. −0.82, *P* = 0. 0021) was statistically significant (Table 3).

**Table 3:** logMAR

Baseline logMAR Visual acuity (SD)	One-week logMAR Visual acuity (SD)	One-month logMAR Visual acuity (SD)	p
−1.6 (0.24)	−0.1 (0.20)	−0.82 (0.10)	0.016

= logarithm of the minimum angle of resolution; SD = standard deviation p = p-value.

The patient with incomplete CRAO and all 11 subjects with subtotal CRAO demonstrated improved vision. The patient with total CRAO didn’t show any change in visual acuity.

### Anatomic Outcome

**Figure 3:**
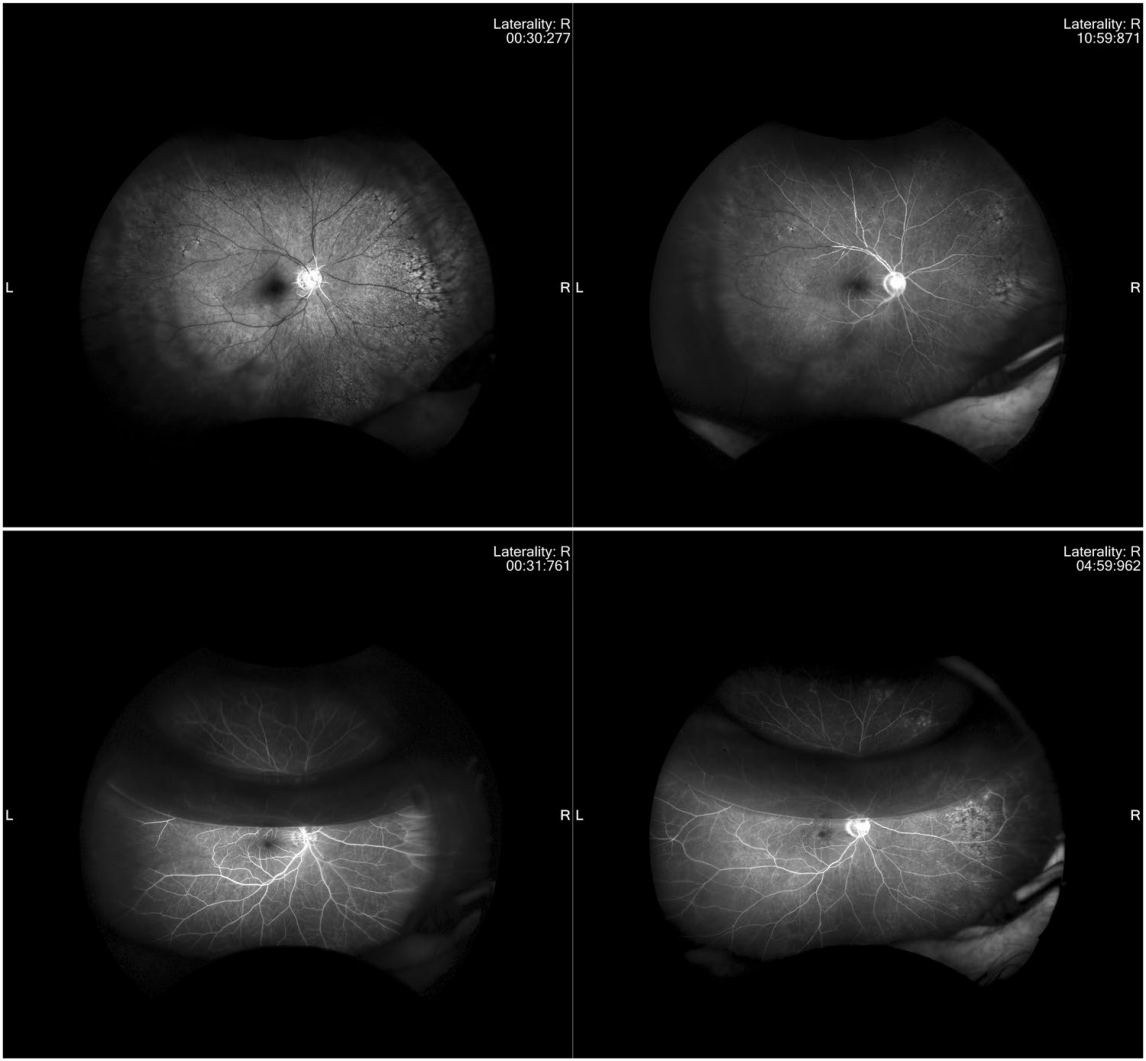
Fluorescein angiography images before and 3 days after cannulation in a patient with CRAO studied. Preoperative visual acuity was counting fingers. None of the arterial vessels filled with dye in the early phase (30 *seconds*) (Upper left). Even in the late phase (*10:59* *seconds*), some of the arterial vessels had not completely filled with the dye (Upper right). All vessels were much more filled with dye, with one–third air after cannulation in early phase (31 *seconds*) (Lower left). All of vessels, including the peripheral vessels, are seen clearly in the late phase (*4:59* *seconds*) (Lower right).

**Figure 4:**
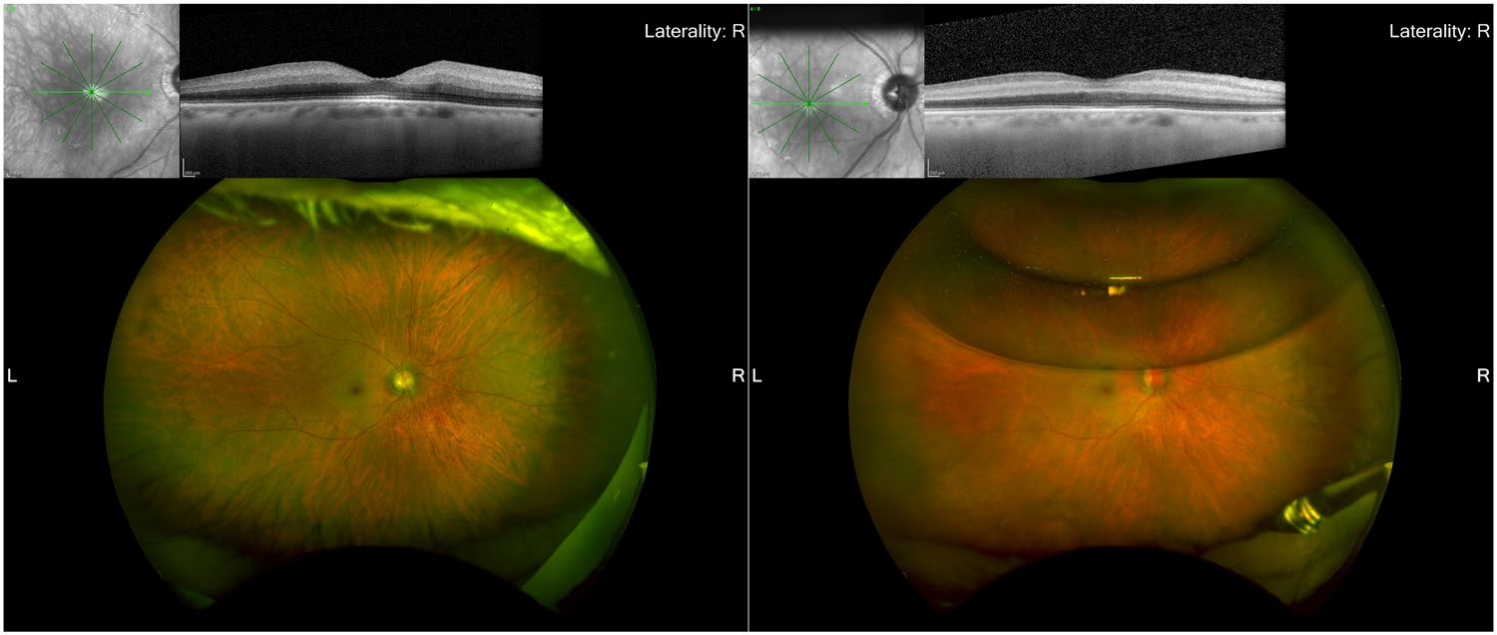
Fundus photographs and optical coherent tomography images before and 3 days after surgery in a patient shown in Fig. 3. A cherry-red spot, narrowed arterial vessels, and mild macular edema are seen and OCT showed thickened inner retinal layer. There was evidence of middle retinal infarction seen, particularly in eyes with incomplete CRAO (Left). Visual acuity was counting fingers. Three days after cannulation, the mild cherry-red spot and clearer retinal vessels are seen with one-third air injected during surgery and OCT showed normal inner retinal layer (Right). Visual acuity was improved 20/400 1 week after surgery.

**Figure 5:**
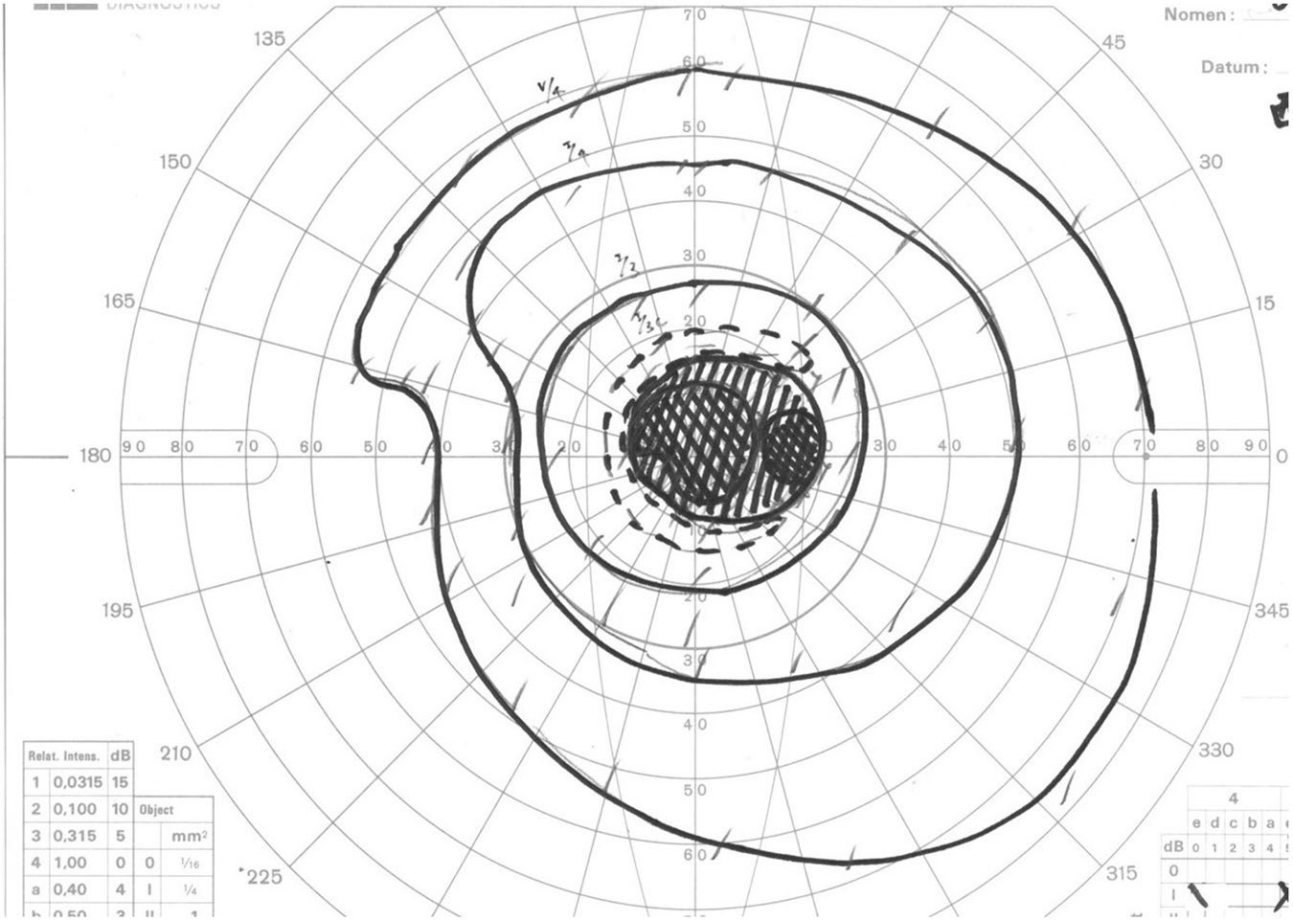
Visual field for this patient one month after surgery. Visual field before cannulation was severely limited. A Goldman visual field examination post-surgery demonstrated much improved visual field, though some relative central scotoma remains.

Preoperative fluorescein angiography showed that the arm-retina times were significantly below normal in all eyes with subtotal and total CRAO (mean time of 56.3 sec.). Normal arm-retina times were seen in the patient with incomplete CRAO. Fluorescein angiography performed 3 days after cannulation revealed complete reperfusion and incomplete reperfusion in 10 eyes and 3 eyes, respectively (Table 2 and Figs 3–5). Preoperative OCT showed slight or mild inner retinal edema in all eyes with subtotal or total CRAO but no edema was present in the eye with incomplete CRAO.

### Surgical Complications

The only serious surgical complication was a postoperative massive vitreous hemorrhage in one eye from the group with subtotal CRAO, and this eye required a second surgery to improve vision.

## Discussion

This is the first report of a study that has assessed the results of retinal arterial cannulation in the treatment of CRAO. The study was performed prospectively in order to determine whether cannulation of a retinal arterial vessel improved both blood circulation and vision. The rate of one-week postoperative visual improvement in 85% of patients treated appears significant enough to support continued use of cannulation, particularly when accompanied by the reperfusion of the retinal vessels noted in this study. There were large postoperative vitreous hemorrhages in one case, which was treated successfully by a second surgery.

Endovascular surgery including cannulation is an effective treatment for diseases with embolization. Because this surgical procedure is challenging, only a few researchers have been able to perform it successfully to date. We recently developed a 47-gauge microneedle made of stainless steel, which is rigid enough to make it possible to approach a tiny structure such as a retinal artery^16^. The development of robotic technology such as the robotic armrest will continue to facilitate performance of the procedure^18^. A robotic armrest has been used successfully in neurosurgery to stabilize surgeon’s hands, and it seems promising for use in retinal surgery^18^. This kind of robotic technology may assist surgeons in performing delicate surgery including retinal cannulation. The cannulation procedure needs to be performed as soon as possible following the onset of symptoms, due to the short survival time of retinal cells in the absence of suitable blood flow^19^. In this study, cannulation was performed within 48 hours in all eyes with CRAO. Although it’s unclear what is an acceptable window for cannulation, the survival of retinal cells is almost certainly dependent on the amount of residual blood flow and the length of time between onset and treatment. Residual blood flow might have been present in eyes in which visual improvement was seen after reperfusion, although it was difficult to clearly detect. The grading scale used in this study hasn’t been widely accepted so it would be necessary in any subsequent studies to develop a method of quantifying blood flow more accurately.

CRAO is caused by the retinal arterial emboli with plaque. Hayreh *et al*. have reported that the carotid artery is the most common source of the plaque leading to CRAO or BRAO, and it can cause retinal arterial occlusion by three mechanisms including embolism, hemodynamic, and serotonin. Embolism is the most common cause, and the major source of the emboli is plaques in the carotid arteries, and much less frequently stenosis. This appears to be well supported by the color Doppler ultrasound images of the internal carotid artery taken in this study, with more than 90% of the CRAO patients exhibiting plaque in the ipsilateral internal carotid artery (Table 2). Theories explain why fibrinolysis with tPA might not be very effective to resolve the primary embolization, because emboli that are mostly composed of cholesterol are less likely to respond to thrombolytic agents such as tPA. One possible benefit of tPA is the ability to dissolve thrombi and plate-fibrin embolic material, and tPA may also assist in dislodging calcific or cholesterol emboli from impaction sites. As well as potential visual functional improvement, reperfusion likely prevents subsequent intraocular neovascularisation. In the procedure used in this study, the embolus was likely pushed back into the internal carotid artery by the force of the microcannulation however there weren’t any related complications, possibly due to the very small size of the thrombus.

The limitations of this study were the relatively small sample size and the fact that the study was conducted at a single center. Moreover, the lack of a control group is a weakness of the study. Comparing results obtained with that of groups that underwent observation only, or cannulation surgery with saline instead of tPA, would help to validate the effects observed. It is important to note that even left untreated, some spontaneous reperfusion would likely occur and in some cases lead to improvement in vision.

In conclusion, further large, multi-center studies are needed to verify the efficacy and safety of this procedure, as well as confirm whether fibrinolytic agents such as tPA can positively influence surgical outcomes. However, the positive results obtained in this study indicate that intra-retinal-arterial cannulation performed shortly after the onset of symptoms of CRAO may improve visual function and microcirculation with few adverse effects.

## Electronic supplementary material

Intra-retinal Arterial Cannulation using a Microneedle for Central Retinal Artery Occlusion
